# Practice patterns in indications, timing, and imaging for clavicle hardware removal: a survey among German-speaking shoulder surgeons

**DOI:** 10.1186/s12891-026-10128-0

**Published:** 2026-06-26

**Authors:** Malik Jessen, Philipp Zehnder, Ahmed Ellafi, Michael Zyskowski, Konstantin Kirchhoff, Peter Biberthaler, Markus Schwarz

**Affiliations:** 1 Department of Sports Orthopedics, TUM University Hospital Rechts der Isar, Ismaninger Str. 22, Munich, 81675 Germany; 2 Department of Trauma Surgery, Klinikum rechts der Isar, Technical University of Munich, TUM University Hospital Rechts der Isar, Ismaninger Str. 22, Munich, 81675 Germany

**Keywords:** Clavicle fracture, Hardware removal, Implant removal, Practice patterns, Timing, Imaging, Return to sport

## Abstract

**Background:**

Clavicle hardware removal is a standard secondary procedure after fracture fixation, yet clinical decision-making regarding indications, timing, imaging, and postoperative recommendations remains highly variable. Despite the frequency of this intervention, no study to date has systematically assessed real-world practice patterns among shoulder and trauma surgeons in the German-speaking region. The purpose of this study was to evaluate current indications, preferred timing, imaging strategies, and postoperative recommendations related to clavicle hardware removal.

**Methods:**

A cross-sectional, web-based survey was distributed to members of the German-speaking Shoulder and Elbow Society (DVSE). The questionnaire assessed surgeon demographics, surgical experience, annual volume of hardware removal, indications for removal (general and absolute), preferred timing, imaging modalities used for preoperative planning, the influence of fracture location, and recommendations for return to sports. Only complete responses were included in the final analysis.

**Results:**

A total of 198 surgeons completed the survey. Irritation (90.9%) and patient preference (87.9%) were the most frequently selected indications for hardware removal, whereas infection (56.6%) was the predominant absolute indication for removal. Most surgeons favored delayed removal: 38.9% recommended hardware removal after more than 18 months, and 42.9% relied on radiographic consolidation as the primary determinant of when to remove the hardware. Radiographs were routinely obtained (99.0%), while CT scans were used selectively (37.4%), particularly for early elective procedures. Most respondents reported no fracture-location–specific differences in removal frequency, although lateral fractures were most cited among those indicating variation. Recommendations for return to sport varied widely, with most surgeons allowing resumption of athletic activity between 6 and 12 weeks postoperatively.

**Conclusion:**

Despite the high volume of clavicle hardware removal procedures, management strategies remain heterogeneous, underscoring persistent uncertainty in indications, timing, imaging protocols, and return-to-sport advice.

**Supplementary Information:**

The online version contains supplementary material available at 10.1186/s12891-026-10128-0.

## Introduction

Clavicle fractures are among the most common injuries of the shoulder girdle and are increasingly treated operatively, particularly in cases of displacement or instability [1–3]. As operative fixation has become more common, so has the frequency of secondary procedures, most notably implant removal [4, 5]. Hardware-related symptoms after clavicle osteosynthesis, such as irritation, prominence, pain, and soft-tissue discomfort, are frequently reported, making the clavicle one of the most common anatomical sites for elective implant removal in orthopedic trauma surgery [6]. Several clinical series report removal rates ranging from 10% to over 50%, depending on the implant type, fracture location, and patient activity level [4, 6–13].

Despite the prevalence of hardware removal, there is no consensus on indications, optimal timing, or the appropriate preoperative workup [6, 10, 14, 15]. Reported indications vary widely across studies, and, in particular, patient-related motivations for hardware removal may differ substantially [10, 14]. While some patients request implant removal because of pain, irritation, implant prominence, or soft-tissue discomfort, others may expect routine or prophylactic removal despite limited symptoms, particularly in young or physically active individuals [10].

Some surgeons also advocate routine or prophylactic implant removal after fracture healing, particularly in young or highly active patients, to prevent future implant-related irritation, mechanical complications, or interference with sports and daily activities [7, 12]. The decision of whether and when to remove implants is further complicated by the absence of validated criteria for radiographic healing, as well as biomechanical considerations regarding the risk of refracture and highly variable patient expectations regarding return to sport or work [7, 16, 17].

Similarly, practice patterns regarding the timing of removal differ substantially [7, 10–14]. While some surgeons advocate for early removal to alleviate symptoms or allow a return to high-demand activities, others recommend delayed removal, typically after 12–18 months, to reduce the risk of refracture [7, 12]. Evidence remains inconclusive, and existing studies are limited by small sample sizes, heterogeneity of implant types, and lack of standardized follow-up [6, 15, 16].

Preoperative imaging strategies also lack standardization. Although radiographs are routinely obtained, the role of CT scanning in confirming union remains controversial [17, 18], and the use of advanced imaging, such as MRI or ultrasound, is seldom reported in the literature [15, 16].

Taken together, the management of clavicle hardware removal is characterized by substantial variability and limited evidence-based guidance, as highlighted by recent cohort studies and systematic reviews [6, 10, 14–16]. To date, no study has systematically evaluated current practice patterns among shoulder and trauma surgeons in the German-speaking region regarding indications, timing, imaging, and postoperative recommendations after hardware removal.

Therefore, the purpose of this study was to assess contemporary practice patterns among members of the German-speaking D-A-CH (Germany, Austria, Switzerland) Shoulder and Elbow Society (DVSE) regarding indications for hardware removal, timing, and imaging strategies used in decision-making.

## Methods

This cross-sectional, web-based survey was conducted among members of the German-speaking D-A-CH Shoulder and Elbow Society (DVSE; D-A-CH: Germany, Austria, Switzerland). Ethics approval and consent to participate. This study was approved by the Institutional Review Board of the Technical University of Munich (TUM) (ethics approval number Ref. 2023-8-S-NP; 17 January 2023). The study was conducted in accordance with the principles of the Declaration of Helsinki. Electronic informed consent was obtained from all participants.

Reporting follows the STROBE guidelines for observational studies and the CHERRIES guidelines for online surveys [19–21]. Completed checklists are provided in the Supplementary Material.

The sampling frame comprised all members of the German-speaking DVSE. The DVSE primarily consists of orthopedic and trauma surgeons with a clinical focus on shoulder and elbow surgery. The survey invitation was distributed via the official DVSE mailing list to approximately 1,395 society members. According to the society administration, only a small number of emails were undeliverable. The survey was open from April 8th, 2025, to July 31st, 2025, with one reminder sent on May 19, 2025. Electronic informed consent was obtained on the landing page; participation was voluntary and anonymous. No IP addresses or identifiers were stored. By design, only complete submissions could be entered (no partial responses). The invitation landing page provided members with information about the study’s purpose, estimated completion time, data usage, and data anonymity. Eligible were clinicians treating clavicle fractures in the D-A-CH region; non-clinicians were not eligible.

### The questionnaire

The questionnaire was developed by the study team based on clinical expertise and prior practice-pattern work, piloted internally, and finalized before distribution. It required approximately five to ten minutes to complete; no incentives were offered. The survey instrument was developed by the authors specifically for this study and was not based on a previously published questionnaire. The full instrument comprised 30 items covering several aspects of clavicle fracture management. In the present analysis, only the items relevant to implant removal after clavicle fracture were included. An English-language translation of the original German version of the complete questionnaire is provided in the Supplementary Material. Items were presented in a fixed order without randomization. All core items were mandatory; respondents could review and change answers before final submission. The survey was administered in German; the whole instrument is provided in the Supplement. The questionnaire covered respondent demographics (discipline, years since board certification, subspecialty training, center type, and annual clavicle fracture volume), imaging and decision thresholds for surgery, and procedure choices by fracture location (nonoperative, plate fixation, intramedullary devices, hook plate/other for lateral fractures). Items were either single-response (mutually exclusive) or multiple-response (“select all that apply”), as indicated in the questionnaire.

Selected items also included an open-ended “Other” field. When a respondent provided only a free-text entry, that response was not counted among the predefined categories for that item; however, the respondent remained part of the overall sample (*n* = 198). Free-text entries were reviewed and thematically grouped; similar answers were aggregated as “Other” and, when sufficiently homogeneous, summarized in the Results and/or Supplement.

For descriptive reporting, we used the number of complete submissions as the denominator (*n* = 198) for all items, including multiple-response items; therefore, percentages for multiple-choice questions may exceed 100%. Figures display absolute counts (n) and the corresponding percentages calculated using *n* = 198 as the denominator, unless stated otherwise. For subgrouped stacked bars, each stack represents 100% within the respective subgroup.

The survey was implemented in Google Forms (Google LLC, Mountain View, CA, USA). Data were exported and managed in Microsoft Excel for macOS, Version 16.99.2 (Microsoft Corp., Redmond, WA, USA).

### Statistical analysis

Data analysis was performed using GraphPad Prism for macOS, Version 10.0.3 (GraphPad Software, San Diego, California, USA) and IBM SPSS Statistics for macOS, Version 30.0.0.0 (IBM Corp., Armonk, New York, USA).

## Results

A total of 198 clinicians completed the survey, corresponding to an approximate response rate of 14.2% among the total number of email recipients, regardless of eligibility. Most respondents were male (86.9%) with a mean age of 47.8 years (Table 1). The majority were board-certified orthopedic or trauma surgeons (91.9%), and 62.6% reported more than 10 years of surgical experience. Over half of the participants (56.6%) reported holding an additional German subspecialty certification. Respondents represented a broad range of practice settings, including university medical centers (15.2%), non-university hospitals (60.1%), and private practices or ambulatory surgery centers (24.7%).

**Table 1:** Participants' demographic data

*Traumanetzwerk-Zentrum der deutschen Gesellschaft für Unfallchirurgie (DGU)*	
	Data
No. of participants	198
Sex	
Male	172 (86.9%)
Female	25 (12.6%)
Diverse	1 (0.5%)
Mean age, years	47.8 ± 10.2 [28- 88]
Years of surgical experience	
Less than 5 years	39 (19.7%)
5 to 10 years	35 (17.7%)
More than 10 years	124 (62.6%)
Current stage of surgical training	
In training for board certification in orthopedic and trauma surgery	16 (8.1%)
Board-certified orthopedic/trauma surgeon without additional subspecialty	16 (8.1%)
Board-certified orthopedic/trauma surgeon in training for subspecialty certification in trauma surgery	24 (12.1%)
Board-certified orthopedic/trauma surgeon in training for subspecialty certification in orthopedic surgery	30 (15.2%)
Board-certified in trauma surgery with additional subspecialty in trauma surgery	101 (51.0%)
Board-certified in orthopedic surgery with additional subspecialty in orthopedic surgery	11 (5.6%)
Country of professional activity	
Germany	172 (86.9%)
Austria	14 (7.1%)
Switzerland	12 (6.1%)
Type of medical institution	
University/tertiary medical center	30 (15.2%)
Non-university medical center	119 (60.1%)
Private practice/ambulatory surgery center	49 (24.7%)
Trauma network affiliation	
Local trauma center	75 (37.9%)
Supraregional trauma center	59 (29.8%)
Not affiliated with a trauma network	64 (32.3%)

Values are *n* (%), age is mean ± SD [range]. Trauma network, local = lokales Traumanetzwerk-Zentrum; supraregional = überregionales

**Fig. 1:**
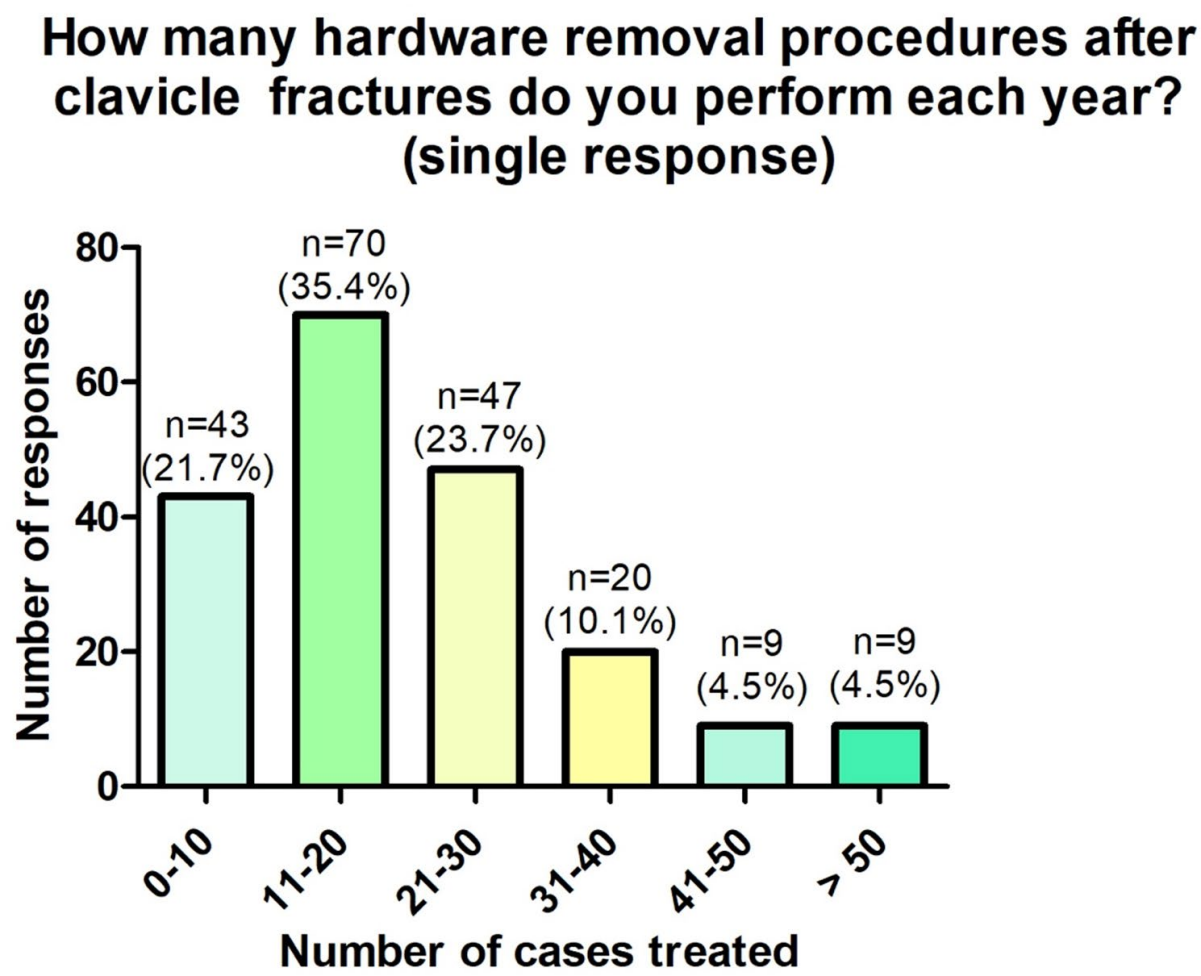
Annual number of hardware removal procedures performed after clavicle fractures (single-response item). Bars indicate the number of respondents (*n*) who performed hardware removal procedures within each annual case-volume category. Percentages beneath each bar are calculated using the total sample (*N* = 198) as the denominator. One answer was permitted

**Fig. 2:**
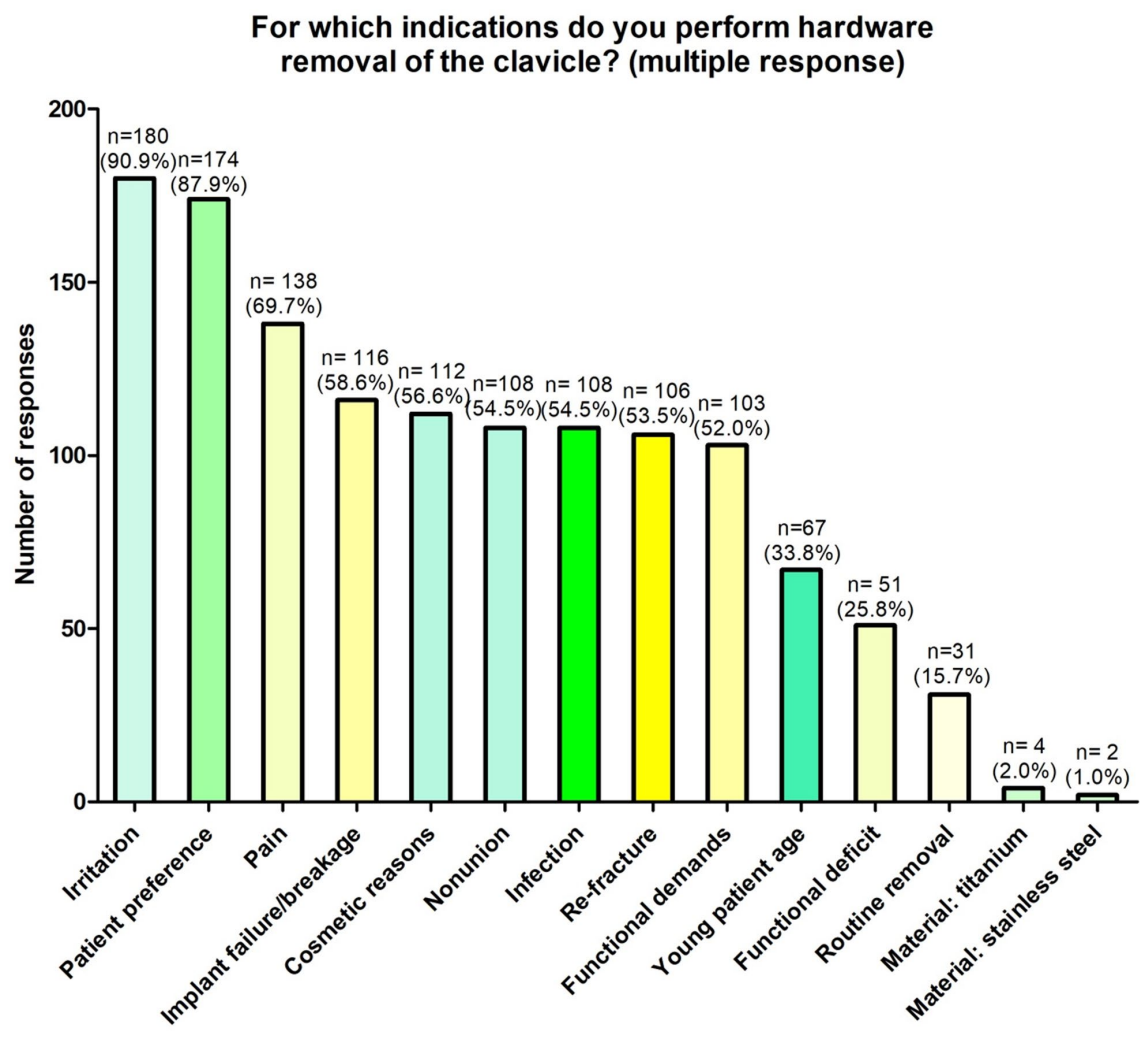
Indications for hardware removal of the clavicle (multiple-response item). Bars display the number of respondents (*n*) selecting each indication for hardware removal. Percentages beneath each bar are calculated using the total survey sample (*n* = 198) as the denominator. Multiple responses were permitted

**Fig. 3:**
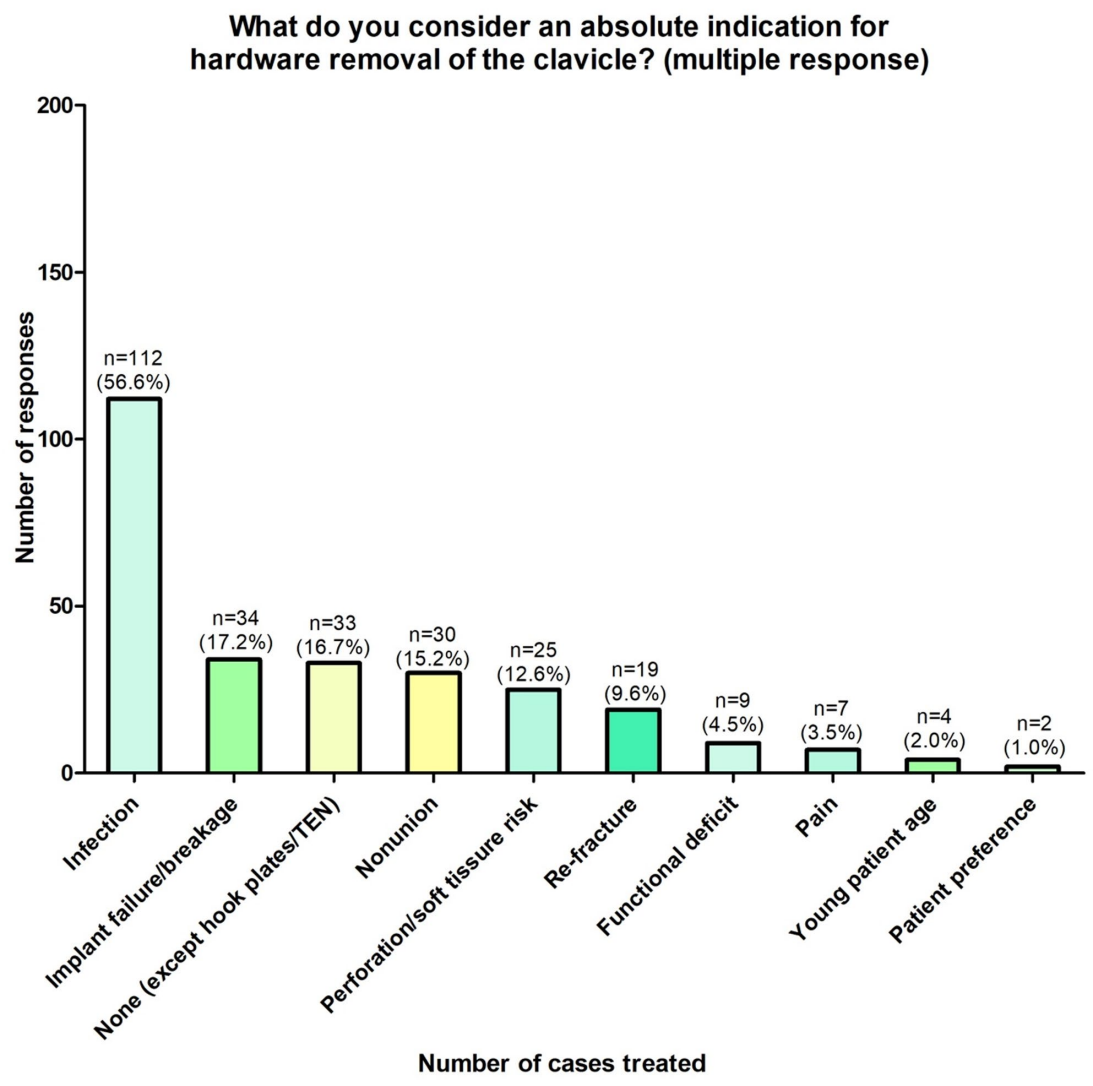
Absolute indications for hardware removal of the clavicle (multiple-response item). Bars indicate the number of respondents (*n*) who identify each factor as an absolute indication for hardware removal. Percentages beneath each bar are calculated using the total survey sample (*n* = 198) as the denominator. Multiple responses were permitted. TEN, titanium elastic nail

Regarding the frequency of hardware removal procedures, most surgeons performed between 11 and 20 removals per year, while fewer than 10% conducted more than 40 annually (Fig. 1). Irritation (90.9%) and patient’s preference (87.9%) were the most frequently selected indications for hardware removal, followed by pain, implant failure or breakage, cosmetic concerns, nonunion, and infection (Fig. 2). Infection was also the most cited absolute indication (56.6%), whereas implant failure, nonunion, perforation, or soft-tissue risk, and re-fracture were chosen less frequently. Patient preference and young age were rarely considered absolute indications (Fig. 3).

**Fig. 4:**
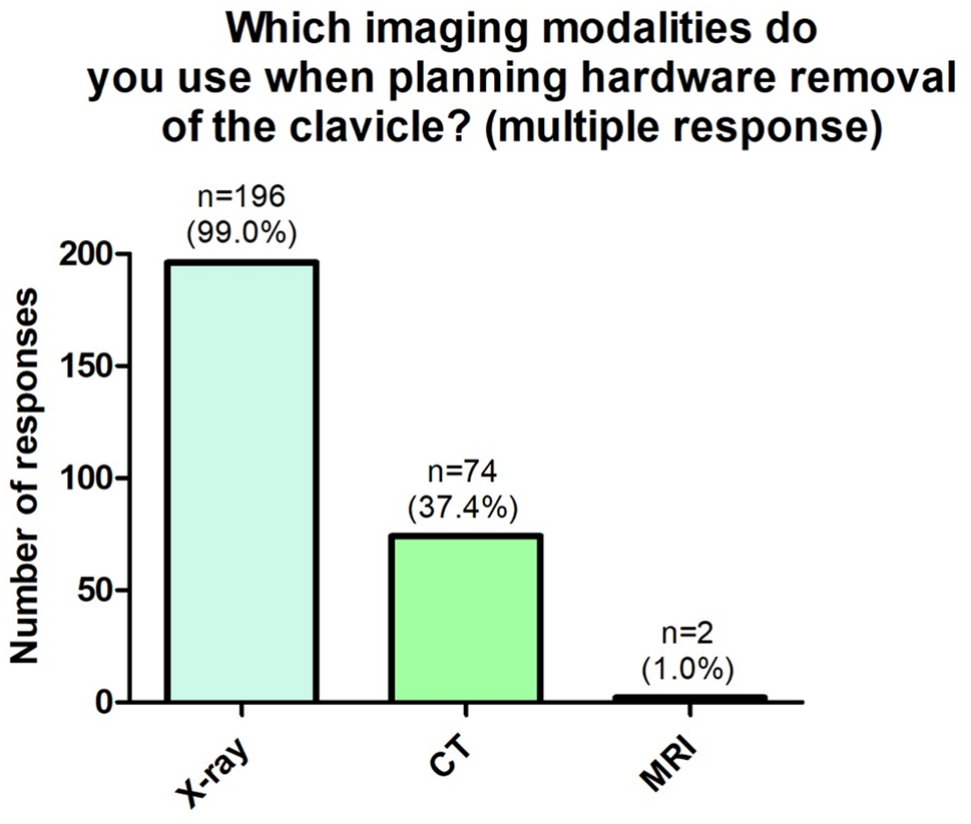
Imaging modalities used when planning hardware removal of the clavicle (multiple-response item). Bars display the number of respondents (n) selecting each imaging modality. Percentages beneath each bar are calculated using the total survey sample (*n* = 198) as the denominator. Multiple responses were permitted

**Fig. 5:**
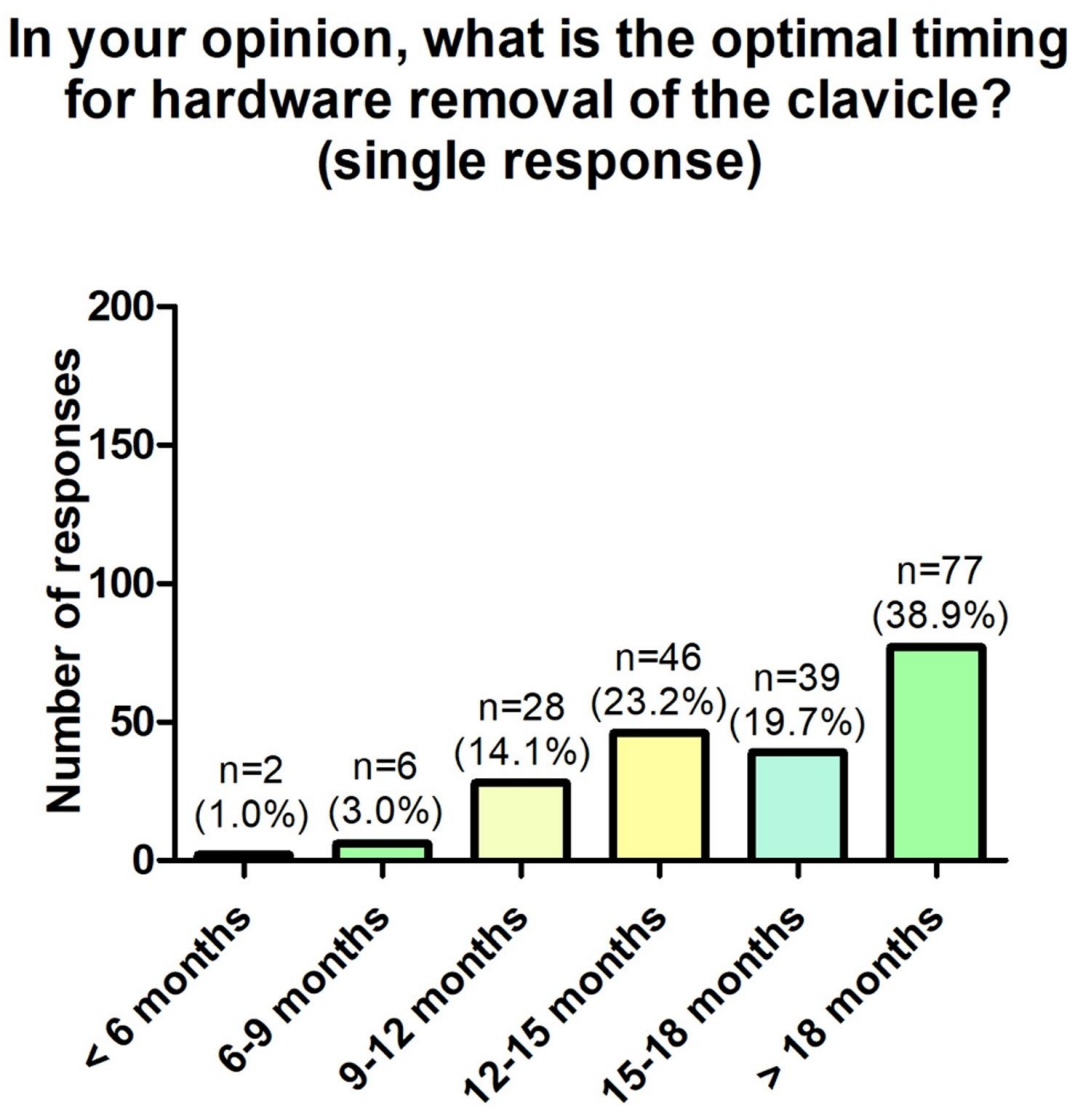
Perceived optimal timing for hardware removal of the clavicle (single-response item). Bars display the number of respondents (n) selecting each time interval as the optimal timing for hardware removal. Percentages beneath each bar are calculated using the total survey sample (*n* = 198) as the denominator. One answer was permitted

**Fig. 6:**
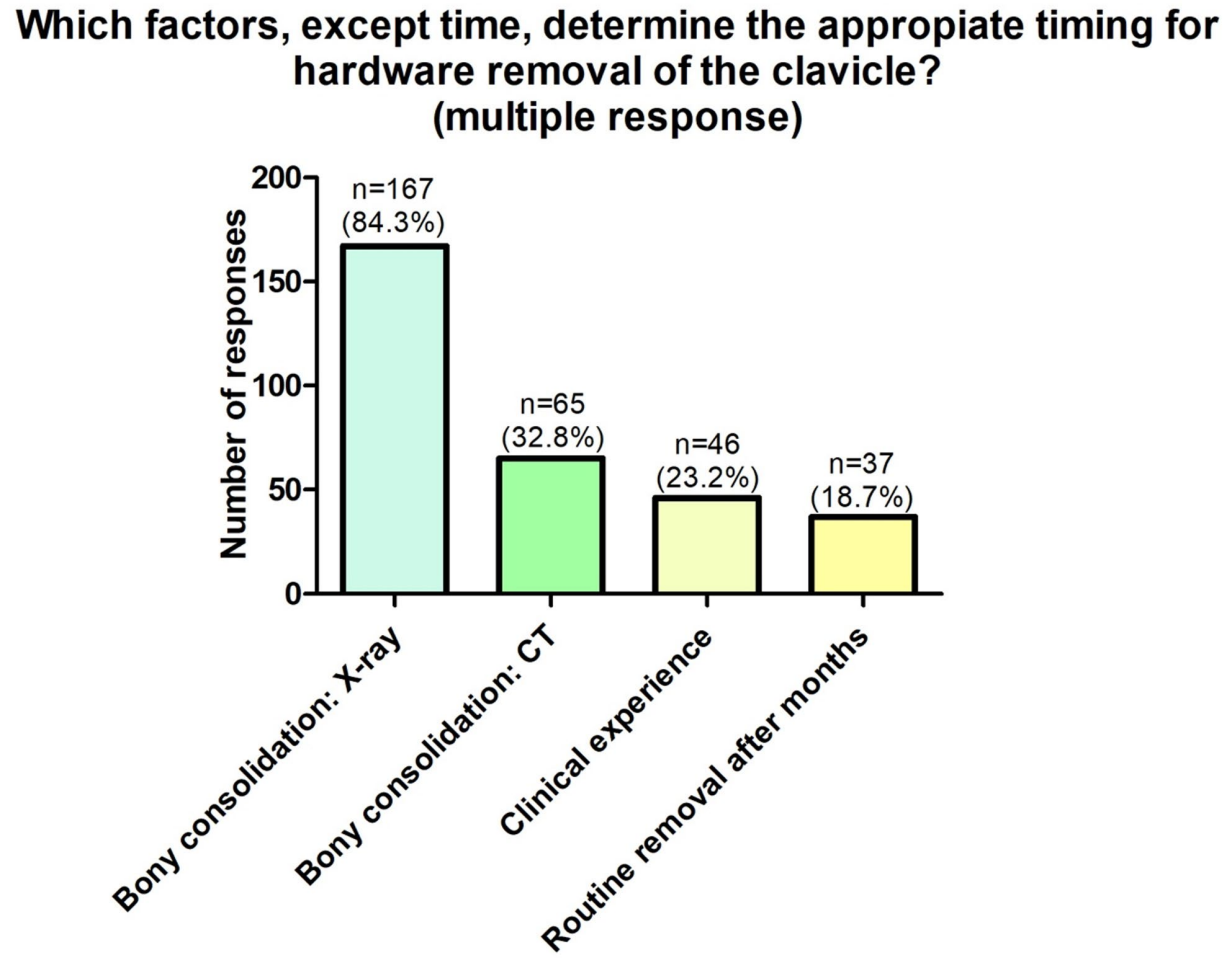
Factors, other than time, that determine the appropriate timing for hardware removal of the clavicle (multiple-response item). Bars display the number of respondents (*n*) selecting each factor. Percentages beneath each bar are calculated using the total survey sample (*n* = 198) as the denominator. Multiple responses were permitted

Standard radiographs were obtained by nearly all respondents (99.0%), whereas CT scans were used by 37.4% and MRI scans by only 1.0% when planning hardware removal (Fig. 4). Most clinicians regarded removal after 18 months as optimal, while fewer favored removal between 12 and 18 months or earlier than 12 months (Fig. 5). The appropriate timing for removal was primarily guided by radiographic evidence of bony consolidation (84.3%), followed by CT findings, clinical experience, and, less commonly, routine time-based protocols (Fig. 6).

**Fig. 7:**
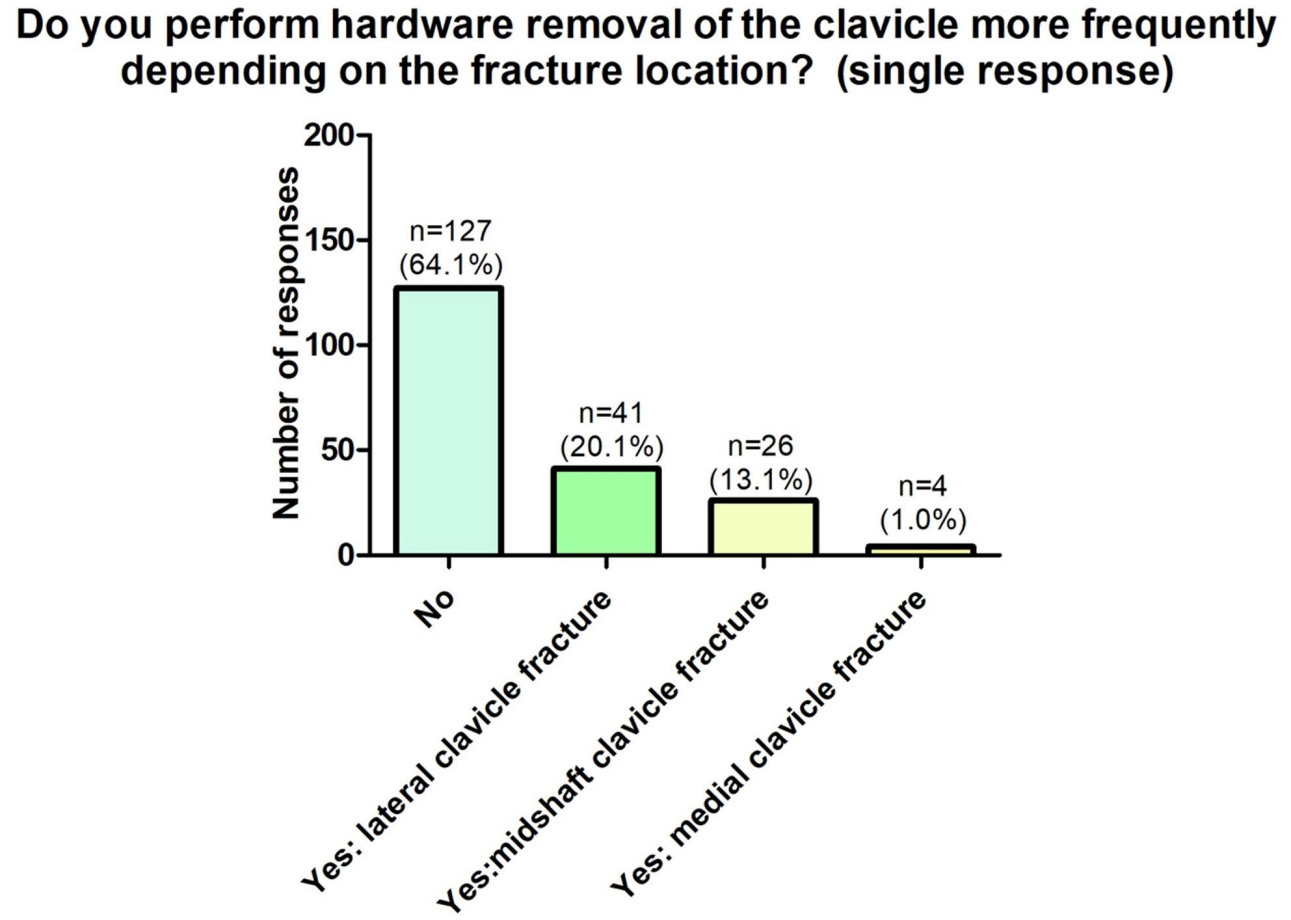
Frequency of hardware removal of the clavicle depending on fracture location (single-response item). Bars display the number of respondents (n) and indicate whether they perform hardware removal more frequently at specific fracture locations. Percentages beneath each bar are calculated using the total survey sample (*n* = 198) as the denominator. One answer was permitted

**Fig. 8:**
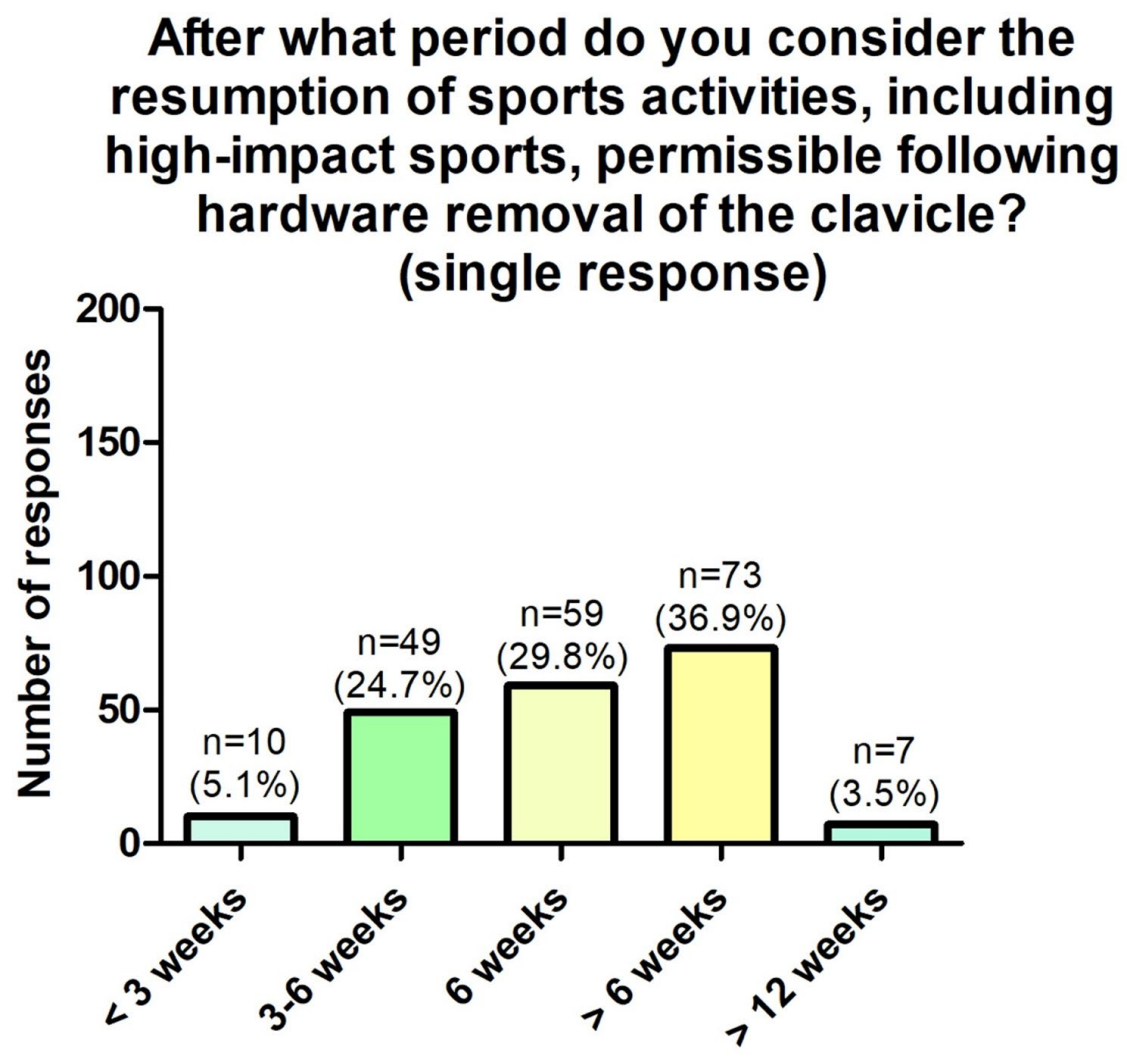
Recommended timing for resumption of sports activities, including high-impact sports, following hardware removal of the clavicle (single-response item)

**Fig. 9:**
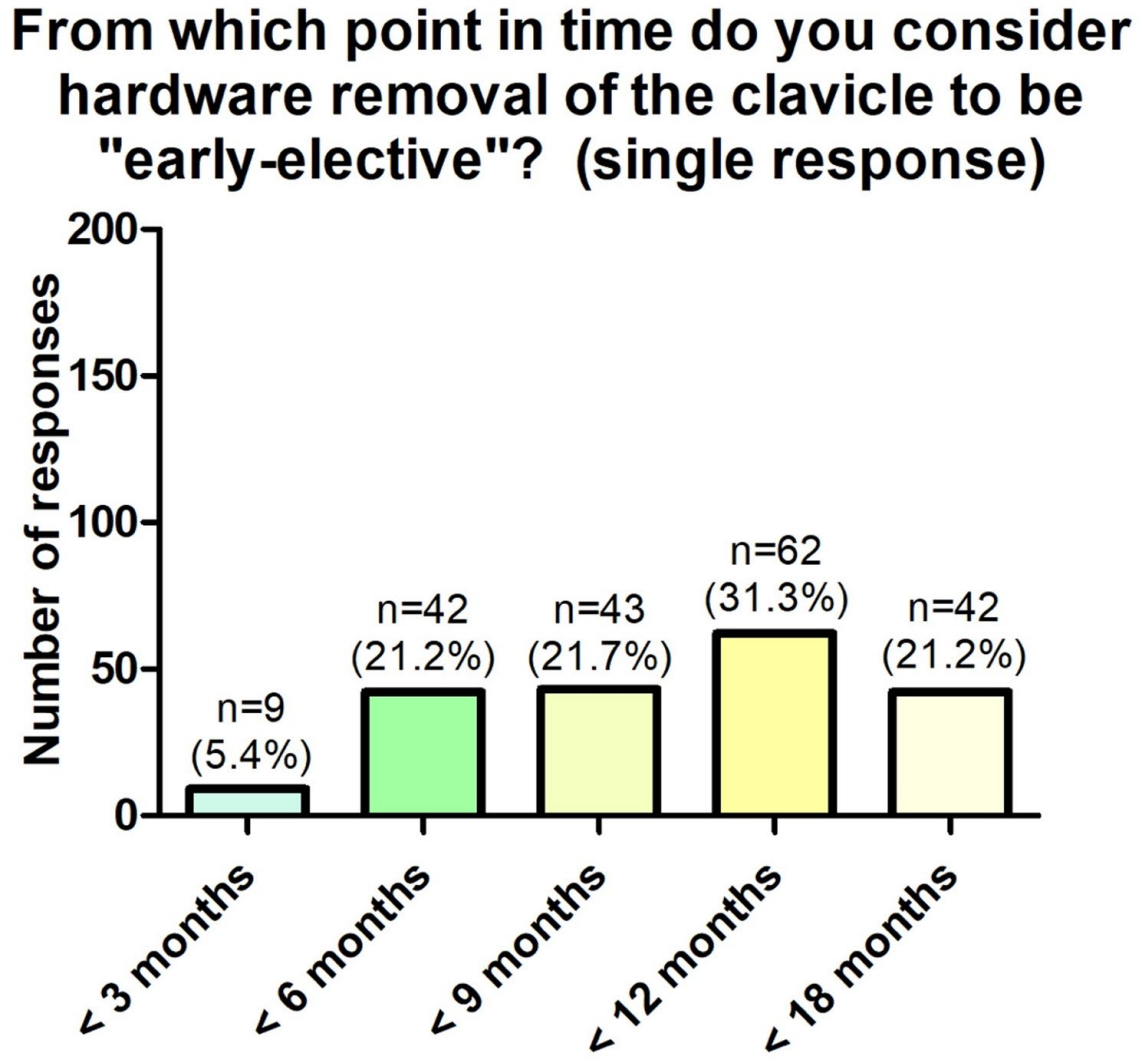
Point in time at which hardware removal of the clavicle is considered “early-elective” (single-response item). Bars display the number of respondents (*n*) selecting each time threshold. Percentages beneath each bar are calculated using the total survey sample (*n* = 198) as the denominator. One answer was permitted

**Fig. 10:**
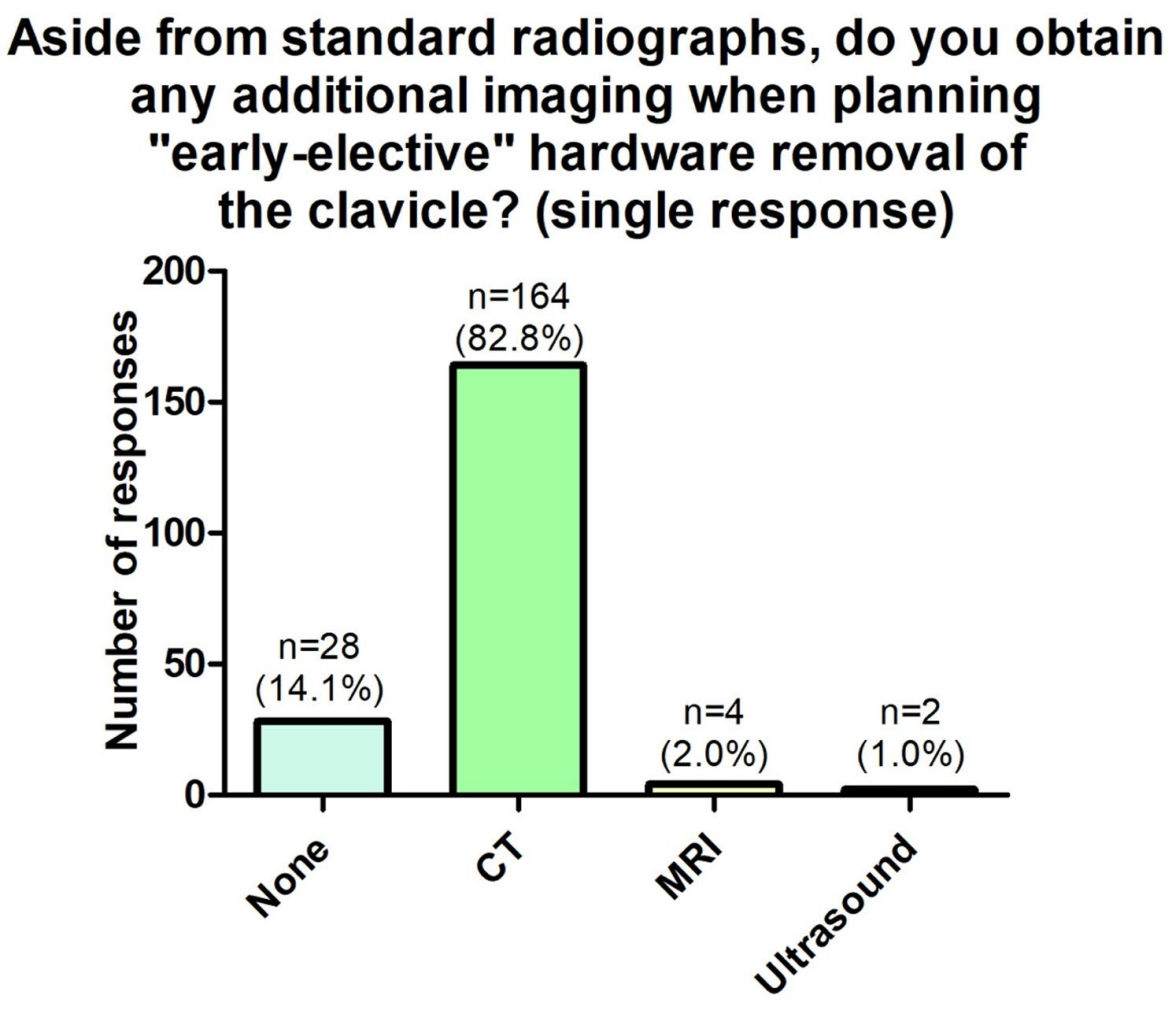
Additional imaging obtained, beyond standard radiographs, when planning early-elective hardware removal of the clavicle (single-response item). Bars display the number of respondents (*n*) selecting each option. Percentages beneath each bar are calculated using the total survey sample (*n* = 198) as the denominator. One answer was permitted

Most respondents reported no difference in the frequency of hardware removal based on fracture location (64.1%). Among those who underwent removal, it was performed most frequently after lateral fractures, followed by midshaft and medial fractures (Fig. 7). Return to sports, including high-impact activities, was generally permitted at 6 weeks (36.9%) or after 6–12 weeks (29.8%), with only a small minority allowing return earlier than 3 weeks (Fig. 8). Definitions of “early-elective” hardware removal varied, most commonly referring to removal within 12 months, while a smaller proportion selected thresholds of 9 or 6 months or less (Fig. 9). When specifically planning early-elective removal, most clinicians obtained additional CT imaging (82.8%), whereas only 14.1% performed no further imaging beyond standard radiographs. MRI (2.0%) and ultrasound (1.0%) were used (Fig. 10).

Bars display the number of respondents (n) selecting each postoperative interval before allowing return to sports. Percentages beneath each bar are calculated using the total survey sample (*n* = 198) as the denominator. One answer was permitted.

## Discussion

This study provides the first comprehensive evaluation of real-world practice patterns regarding indications, timing, imaging, and postoperative recommendations for clavicle hardware removal among shoulder and trauma surgeons in the German-speaking D-A-CH region. The results demonstrate considerable heterogeneity in clinical decision-making, reflecting the limited evidence and lack of consensus previously described in the literature. Across all domains assessed—indications, timing, imaging, and postoperative guidance—substantial variability was observed, underscoring the need for more precise, evidence-based recommendations.

Consistent with prior clinical series reporting irritation, prominence, and soft-tissue discomfort as the most common drivers of implant removal [4, 6, 10–14], the overwhelming majority of respondents in our cohort endorsed irritation (90.9%) and patient preference (87.9%) as indications for removal.

In addition, neuropathic pain related to supraclavicular nerve irritation or injury may represent an important contributor to persistent postoperative symptoms and patient dissatisfaction following clavicle fixation [22, 23].

Among the surveyed surgeons, infection was the most frequently selected absolute indication for implant removal (56.6% of respondents), a finding consistent with established principles of fracture-related infection management [24]. This percentage does not reflect the incidence of postoperative infection after clavicle fixation, but rather the proportion of respondents who identified infection as an absolute indication for implant removal. Importantly, indications such as infection or nonunion should be interpreted as reasons for implant removal driven by complications rather than purely elective procedures. In these situations, implant removal or revision is often required as part of the overall management strategy for fracture-related complications.

Notably, patient preference and young age were rarely considered absolute indications. This discrepancy highlights a fundamental challenge: while many patients request implant removal, surgeons do not uniformly consider such requests mandatory. The “symptomatic but not absolute” indication category thus remains clinically ambiguous and may benefit from the development of standardized criteria.

The presented findings reveal a broad range of preferred removal timing. However, the plurality of respondents favored a delayed approach, with 38.9% selecting an interval of more than 18 months as the optimal choice. This is consistent with concerns about the risk of refracture following premature removal. Accordingly, decisions regarding delayed implant removal are often influenced not only by chronological time intervals but also by radiographic healing, perceived biomechanical stability, patient activity level, and surgeon experience [7, 16–18].

In addition, delayed removal is often advocated to allow more complete osseous remodeling and reduction of stress riser effects caused by residual screw holes after plate removal [25]. Several recent retrospective studies support delayed removal, with refracture rates ranging from 2% to 7%. Earlier removal, shorter working length, or residual screw-hole stress risers increase the risk of refracture [7, 16, 18]. The extensive cohort analysis by Kessler et al. found that a shorter plate working length was independently associated with a significantly higher risk of refracture after removal [7], reinforcing the premise that implant biomechanics should guide surgical decision-making. Nevertheless, some studies have suggested that patients may benefit from earlier removal, such as to relieve irritation or return to sport, without definitive evidence that this increases complications [10]. Taken together, the current literature yields variable results, which explains the wide variation observed in our survey.

Although almost all respondents (99.0%) obtained radiographs, only one-third routinely used CT scans to confirm bony union prior to elective removal. The limited use of CT contrasts with the ongoing debate about its diagnostic superiority. Nicholson et al. demonstrated that CT identifies incomplete union more accurately than conventional radiographs and that approximately one in five suspected clavicle nonunions ultimately represent delayed union rather than true nonunion [17]. Nevertheless, the routine application of CT remains controversial due to radiation exposure, cost, and unclear impact on clinical outcomes. Advanced imaging modalities such as MRI or ultrasound were rarely selected in our cohort, consistent with previous reports suggesting limited clinical utility for implant removal planning [15, 16]. When considered, MRI use is likely influenced by implant-related artifact limitations and may therefore depend on the specific implant material. Notably, CT use increased sharply when surgeons specifically planned early elective removal, suggesting that imaging strategies may be context-dependent.

Although most respondents reported no location-specific variation in removal rates, those who did identified lateral fractures as the most frequent. This aligns with known biomechanical challenges of lateral clavicle fixations and higher implant irritation rates reported for hook plates and lateral locking plates [12].

Implant-related symptoms and the need for hardware removal may differ substantially depending on the fixation technique. Plate fixation, particularly in the subcutaneous anatomy of the clavicle, is frequently associated with implant prominence and soft-tissue irritation, whereas intramedullary devices may present different complication profiles and considerations regarding implant removal [6, 12, 13].

Postoperatively, most surgeons allowed return to sports, including high-impact activities, between 6 and 12 weeks.

Several series report that following operative fixation of displaced midshaft clavicle fractures, up to 94% of athletes resume sports at their pre-injury level within 2–3 months [26–28]. However, these data refer to outcomes after ORIF — not after subsequent implant removal. To date, no published study has systematically evaluated the timing of return to sport or the risk of re-fracture following elective hardware removal.

Thus, the variability seen in our survey likely reflects clinical experience rather than evidence-based mandates. In addition, return-to-sport recommendations and decisions regarding elective implant removal may vary depending on the specific sport and the risk of recurrent trauma or high-energy falls, particularly in contact or high-impact activities such as cycling, motorcycling, or ice hockey.

Across all domains explored, our findings align with the broader literature, which describes implant removal as a procedure lacking standardized guidelines and characterized by surgeon-dependent decision-making [6, 10, 14–16]. Hardware removal rates differ significantly across implant types; for instance, dual plating has recently been shown to reduce irritation and removal rates compared with single plating [29, 30]. These innovations highlight how implant evolution may impact future practice patterns, although long-term data remain limited.

Several limitations must be considered. First, this study was survey-based and descriptive; therefore, the results reflect self-reported surgeon opinions and practice patterns rather than patient-level clinical outcomes. Consequently, no conclusions can be drawn regarding complication rates, functional outcomes, refracture risk, or the clinical superiority of specific strategies. Second, the questionnaire was developed specifically for this study and was not a previously validated instrument, which may limit the reliability and reproducibility of the findings. Third, the survey was limited to members of the German-speaking D-A-CH Shoulder and Elbow Society (DVSE). This may have introduced selection bias toward shoulder-focused surgeons and may not fully reflect practice patterns among all trauma surgeons treating clavicle fractures outside the DVSE network.

Similarly, the reported annual volume of hardware removal procedures does not necessarily reflect the overall operative treatment rate for clavicle fractures but rather likely reflects the referral patterns and case volume of specialized shoulder and trauma surgeons participating in the survey.

As a further result, the findings may not be generalizable to surgeons practicing in other countries, healthcare systems, or training environments.

The present study should be interpreted as a descriptive assessment of current surgeon-reported practice patterns rather than as outcome-based evidence for specific management strategies. While the findings do not establish clinical superiority or infer causality, they provide insight into the substantial variability in clinical decision-making regarding clavicle hardware removal across the D-A-CH region. This heterogeneity may help identify areas where future prospective studies and consensus recommendations are needed.

## Conclusion

Despite the high volume of clavicle hardware removal procedures, management strategies remain heterogeneous, underscoring persistent uncertainty in indications, timing, imaging protocols, and return-to-sport advice.

## Supplementary Information

Supplementary Material 1.

Supplementary Material 2.

Supplementary Material 3.

## Data Availability

The datasets generated and analysed during the current study are available from the corresponding author on reasonable request.
