# Supplementary material for: Practice patterns in indications, timing, and imaging for clavicle hardware removal: a survey among German-speaking shoulder surgeons
Source: BMC Musculoskelet Disord. 2026 Jun 26;27:557. doi: 10.1186/s12891-026-10128-0 (PMC13321908; doi:10.1186/s12891-026-10128-0)
Supplement: Supplementary file 3 — Supplementary Material 3. [file 12891_2026_10128_MOESM3_ESM.docx]

**Supplementary: English-language translation of the questionnaire used in the clavicle fracture survey**

Note: **The original questionnaire was distributed in German to participants in the D-A-CH region. The version provided here is an English-language translation prepared for journal transparency and supplementary documentation purposes.**

Unless otherwise indicated, a single response was permitted. Items labeled "Multiple responses possible" allowed multiple answers.

1. Please select your sex.

- Male

- Female

- Diverse

2. How old are you?

Open text response: _______

3. In which country do you work?

- Germany

- Austria

- Switzerland

4. Where do you work?

- Non-university hospital

- University hospital

- Private practice / private clinic

5. Which care-level category does your hospital belong to?

- Local trauma center

- Supraregional trauma center

- Not affiliated with a network

6. What is your current stage of training?

- Training toward board certification in orthopaedic and trauma surgery

- Board-certified specialist

- Board-certified specialist in training for the additional qualification in special trauma surgery

- Board-certified specialist in training for the additional qualification in special orthopaedic surgery

- Additional qualification in special trauma surgery completed

- Additional qualification in special orthopaedic surgery completed

- Other: __________

7. How much operative professional experience do you have (since obtaining board certification in orthopaedic and trauma surgery or board certification in general surgery with a focus on trauma surgery)?

- Less than 5 years

- 5-10 years

- More than 10 years

8. How many clavicle fractures do you treat nonoperatively per year?

- 0-10

- 11-20

- 21-30

- 31-40

- 41-50

- More than 50

- Other: ______

9. At what degree of displacement, expressed as a percentage of shaft width, do you indicate surgery for midshaft clavicle fractures?

- 25%

- 50%

- 75%

- 100%

- More than 100%

- Other: ______

10. At what degree of displacement, expressed as a percentage of shaft width, do you indicate surgery for medial clavicle fractures?

- 25%

- 50%

- 75%

- 100%

- More than 100%

- Other: ______

11. In the case of a medial clavicle fracture, which factors do you consider additional indications or surgery? (Multiple responses possible)

- Young patient age

- Number of fragments

- Multiple fractures of the SSSC (Superior Shoulder

Suspensory Complex)

- Other: ______

12. Which osteosynthesis material do you use for the operative treatment of a simple transverse fracture? (Multiple responses possible)

- Reconstruction plate (steel)

- Locking Compression Plate (LCP)

- Anatomically precontoured plate

- TEN

- Other: ______

13. How many clavicle fractures do you treat with plate osteosynthesis per year?

- 0-10

- 11-20

- 21-30

- 31-40

- 41-50

- More than 50

- Other: ______

14. If a plate is used: How many screws do you use on each side of the fracture? (Multiple responses possible)

- 1

- 2

- 3

- 4

- 5

- 6

- Other: ______

15. How many clavicle fractures do you treat with an elastic stable intramedullary nail (ESIN or TEN) per year?

- 0-10

- 11-20

- 21-30

- 31-40

- 41-50

- More than 50

- Other: ______

16. In the case of a lateral clavicle fracture, which fractures according to the Jäger & Breitner classification do you treat surgically? (Multiple responses possible)

- Type 1 according to Jäger/Breitner

- Type 2a according to Jäger/Breitner

- Type 2b according to Jäger/Breitner

- Type 3 according to Jäger/Breitner

- Type 4 according to Jäger/Breitner

- Other: ______

17. In the case of a lateral clavicle fracture, which system should currently be considered the gold standard in your opinion?

- K-wires

- Plate osteosynthesis without acromioclavicular-joint stabilization, for example using the DogBone ™ technique

- Plate osteosynthesis with acromioclavicular-joint stabilization, for example using the DogBone ™ technique

- Hook plate

- Other: ______

18. How many clavicle fractures do you treat with a hook plate per year?

- I generally do not use hook plates

- 1-10

- 11-20

- 21-30

- 31-40

- 41-50

- More than 50

- Other: ______

19a. In the case of a lateral clavicle fracture, do you always consider vertical acromioclavicular joint (ACJ) instability an indication for surgery?

- Yes

- No

- Other: ______

19b. In your opinion, what constitutes an indication for additional coracoclavicular (CC) ligament reconstruction in the surgical treatment of a lateral clavicle fracture with vertical ACJ instability? (Multiple responses possible)

- Young patient age

- Degree of fracture displacement

- Number of fragments

- Isolated superior displacement of the lateral clavicle

- Other: ______

20. How many implant removals after clavicle osteosynthesis are performed per year in total at your hospital/practice clinic?

- 0-10

- 11-20

- 21-30

- 31-40

- 41-50

- More than 50

- Other: ______

21. For which indications do you perform implant removal at the clavicle? Please select all applicable responses.

- Routine removal

- Irritation

- Infection

- Patient preference

- Patient age

- Pain

- Cosmetic reasons

- High functional demands (e.g., athletes, physically demanding occupation)

- Pseudarthrosis

- Implant material: titanium

- Implant material: stainless steel

- Functional deficit

- Refracture

- Implant failure / breakage

- Other (specified in Question 22)

22. For which reasons not listed in Question 21 do you perform implant removal

Open text response: _______

23. In your opinion, what constitutes an absolute indication for implant removal at the clavicle?

Open text response: _______

24. Do you perform implant removal more frequently depending on fracture location?

- No

- Yes, more frequently after lateral clavicle fractures

- Yes, more frequently after midshaft clavicle fractures

- Yes, more frequently after medial clavicle fractures

25. In your opinion, what is the optimal timing for implant removal at the clavicle?

- Less than 6 months

- 6-9 months

- 9-12 months

- 12-15 months

- 15-18 months

- More than 18 months

- Other: ______

26. Which imaging modality do you use for planning implant removal? (Multiple responses possible)

- Radiographs

- CT

- MRI

- Other: ______

27. On what factors do you base the timing of implant removal at the clavicle? (Multiple responses possible)

- Radiographic evidence of bony union

- CT-based evidence of bony union

- Personal experience

- Routine removal after xx months

- Other: ______

28. After what period of time do you consider return to sports activities—particularly high-load sports such as handball, tennis, or mountain biking—to be permitted again after implant removal at the clavicle?

- <3 weeks

- 3-6 weeks

- 6 weeks

- >6 weeks

- Other: ______

29. From what time point onward would you consider implant removal at the clavicle to be early elective?

- Less than 3 months

- Less than 6 months

- Less than 9 months

- Less than 12 months

- Less than 18 months

30. Apart from radiographs, do you perform any additional imaging for planning early elective implant removal? (Multiple responses possible)

- CT

- MRI

- Other: ______
